# Identification and characterization of novel chikungunya virus polymerase inhibitors

**DOI:** 10.1128/jvi.00305-26

**Published:** 2026-05-19

**Authors:** Peiqi Yin, Ryan Boyce, Sainan Wang, Michael Sirrine, Alexander Leach, Dillon Chu, Dariia Vyshenska, Zafer Sahin, Jenny Wong, Tahirah Moore, Devin Shane M. Lewis, Stephen C. Pelly, Dennis Liotta, Andres Merits, Alexander L. Greninger, Richard K. Plemper, Margaret Kielian, Robert M. Cox

**Affiliations:** 1 Department of Cell Biology, Albert Einstein College of Medicine 2006, Bronx, New York, USA; 2 Center for Translational Antiviral Research, Institute for Biomedical Sciences, Georgia State University 1373, Atlanta, Georgia, USA; 3 Institute of Bioengineering, University of Tartu, Tartu, Estonia; 4 Virology Division, Department of Laboratory Medicine and Pathology, University of Washington 7284, Seattle, Washington, USA; 5 Department of Chemistry, Emory University 1371, Atlanta, Georgia, USA; University of North Carolina at Chapel Hill, Chapel Hill, North Carolina, USA

**Keywords:** nsP4 inhibitor, antiviral, chikungunya

## Abstract

Chikungunya virus (CHIKV) and other alphaviruses in the *Togaviridae* family are positive-sense RNA viruses and major human pathogens, causing millions of infections worldwide. In humans, alphaviruses, such as CHIKV, Mayaro, and Ross River viruses, typically cause arthritogenic disease characterized by debilitating arthralgia, joint inflammation, fever, and rash. Although a vaccine was recently approved for use against CHIKV, no vaccines are licensed against other alphaviruses. No antiviral treatments are available to prevent or treat infections by any alphavirus. To address this unmet need, we used a CHIKV nanoluciferase reporter virus to develop a high-throughput screening assay for novel small-molecule inhibitors. From this campaign, we identified several unique inhibitors of CHIKV replication. Mechanistic characterization of two inhibitors suggests that both target the nsP4 RNA-dependent RNA polymerase. Susceptibility profiling pinpointed unique nsP4 mutations that specifically conferred resistance but also decreased viral fitness in competition studies. *In silico* docking and resistance profiling analyses indicated potential binding sites of the inhibitors near the polymerase active site. Collectively, these results define multiple chemotypes for further development and highlight novel molecular targets within nsP4 for CHIKV inhibition.

**IMPORTANCE:**

Chikungunya virus is a mosquito-borne pathogen that has caused millions of human infections worldwide, producing severe fever, rash, and long-lasting joint pain that can persist for months. Related viruses, such as Mayaro and Ross River viruses, also cause debilitating disease, yet no antiviral drugs are available to treat any infection caused by this family of viruses. In this study, we developed a high-throughput assay that allowed us to rapidly identify compounds capable of blocking chikungunya virus replication. We discovered new hit compounds that inhibit virus growth. Our results suggest that the two most promising hit candidates target the viral nsP4 polymerase. By identifying these novel inhibitors and characterizing both their mechanisms of action and resistance profiles, we have established the groundwork for future efforts to develop much-needed therapies against chikungunya virus and related pathogens.

## INTRODUCTION

Alphaviruses are enveloped, single-stranded, positive-sense RNA viruses that infect humans worldwide (1). They are primarily transmitted by mosquitoes, with small mammals and birds acting as reservoirs in nature (2, 3). Alphaviruses are grouped into encephalitic or arthritogenic based on their typical disease symptoms in humans. The arthritogenic alphaviruses include chikungunya virus (CHIKV), Mayaro virus (MAYV), o’nyong-nyong virus (ONNV), and Ross River virus (RRV) and generally cause disease characterized by fever, rash, joint pain, and arthralgia, which can persist for months (3–5). Severe cases can occasionally lead to neurological complications, fever, and death (6, 7). Encephalitic alphaviruses such as Venezuelan equine encephalitis virus (VEEV), western equine encephalitis virus (WEEV), and eastern equine encephalitis virus (EEEV) more commonly present with febrile illness and severe neurological disease, including life-threatening encephalitis (8). For EEEV, mortality rates can reach 50%, with sporadic outbreaks documented in the eastern United States (9).

CHIKV originated in sub-Saharan Africa and has recently spread across the globe, becoming endemic in many regions of Central America, the Caribbean, South America, Africa, and Southeast Asia (4, 10). The first documented case of CHIKV in the Americas occurred in 2013 on St. Martin Island, and over a million cases have been reported since (11). CHIKV imposes a significant health burden on millions of people, in particular since acute infections can progress to chronic, debilitating polyarthritis, with symptoms persisting for months or years (12). The spread of CHIKV is linked to the expanding distribution of mosquito vectors, the consequences of climate change, and to viral mutations that enhance transmission efficiency by these vectors (4, 10, 13). CHIKV is spread by both *Aedes aegypti* and *Aedes albopictus* mosquitoes, and current models predict that hundreds of millions more people could be at risk of CHIKV infection by the end of the 21st century (14–16). Recent outbreaks in China, France, and other previously unaffected regions underscore the rapidly expanding distribution of CHIKV (17, 18). Two vaccines, Vimkunya and Ixchiq, were recently approved in the United States (3). However, the FDA subsequently withdrew approval for the Ixchiq vaccine due to serious adverse events, including several deaths. Currently, there are no licensed antiviral therapies against CHIKV, and treatment is limited to nonsteroidal anti-inflammatory drugs (19) and supportive care.

Direct-acting small-molecule antivirals are a promising strategy for treating viral infections and could offer a stable and rapid adjunct to vaccination in controlling CHIKV outbreaks. CHIKV and other alphaviruses infect cells by receptor-mediated endocytosis and low-pH-triggered fusion in acidic endosome compartments (1). The RNA genome is thereby released into the cytoplasm, uncoated, and translated to generate the membrane-associated replication complex (20, 21). This complex is generated from a nonstructural (ns) polyprotein (P1234), which is processed by the essential nsP2 protease to produce nsP1-4 (1). Among these proteins, the nsP2 protease activity and the nsP4 RNA-dependent RNA polymerase (RdRp) are especially attractive targets for inhibition. Numerous strategies have been explored to inhibit nsP2, including peptidomimetics, cysteine-reactive “warheads,” and allosteric inhibitors (22–24). However, these efforts have yet to yield a hit candidate that has advanced to clinical use. The nsP4 RdRp activity is also a major target for small-molecule inhibitors, especially since nsP4 is highly conserved among alphaviruses (25). Several nucleoside analogs potently inhibit alphavirus replication, including favipiravir (26), ribavirin (27), β-D-N4-hydroxycytidine (NHC), the bioactive metabolite of molnupiravir (27), sofosbuvir (28), and 4′-fluorouridine (4′-FlU, EIDD-2749) (29–31). To date, however, none have been licensed for clinical use. A drawback to nucleoside analog therapies is their inherent potential for toxicity through incorporation by host cell polymerases, which is especially significant when treating neonatal populations, a group that, along with immunosuppressed patients and the elderly, is particularly susceptible to severe CHIKV disease. Allosteric inhibitors of nsP4 could therefore represent an important strategy for safer, more targeted antiviral therapies, but to date, no small-molecule allosteric inhibitors of CHIKV nsP4 have been identified.

Here, we conducted high-throughput screening (HTS) using a CHIKV nanoluciferase reporter virus followed by an extensive counter-screening campaign. We identified several hit compounds with anti-CHIKV activity and demonstrated that they were also active in CHIKV replicon assays. Further studies of two hit compounds showed that they inhibited CHIKV replication in normal human dermal fibroblasts and CHIKV production in human cells. Both compounds showed inhibition against a subset of alphaviruses in trans-replicase assays. Resistance profiling revealed that mutations in nsP4 reduce viral susceptibility to these compounds without increasing viral fitness. Resistance profiling and *in silico* docking suggest interactions with two previously uncharacterized sites in the viral nsP4 polymerase. Collectively, these findings reveal new anti-alphavirus chemotypes, likely targeting CHIKV nsP4, providing a foundation for future development of these small-molecule polymerase inhibitors.

## RESULTS

### HTS campaign

**Fig 1:**
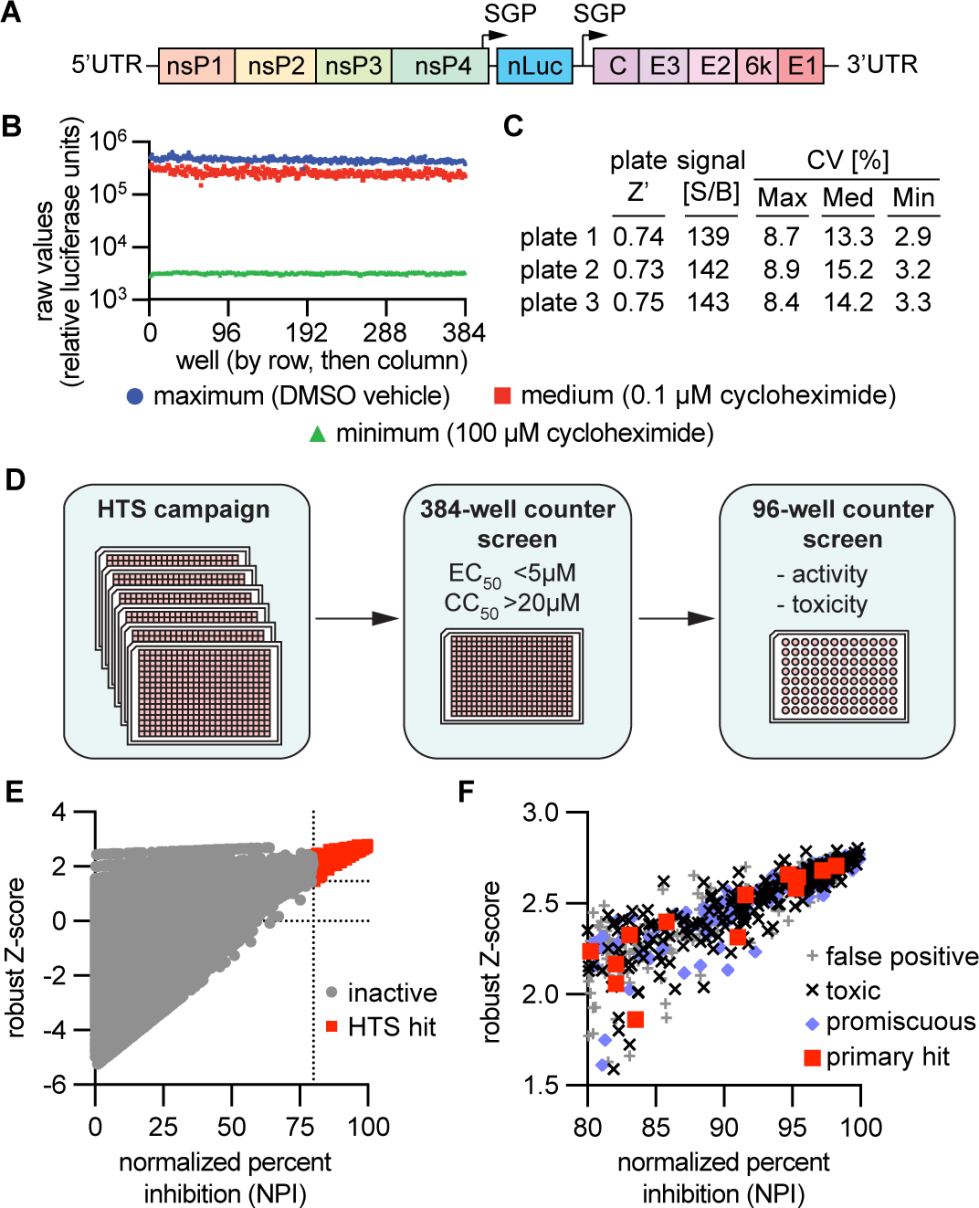
CHIKV HTS campaign. (**A**) Diagram of the recombinant CHIKV reporter virus (CHIKV-nLuc) expressing nanoluciferase from the viral subgenomic promoter (SGP) and structural proteins from an additional SGP inserted between the nLuc and capsid ORFs that was used to develop an HTS assay. (**B**) Validation of automated HTS protocol in 384-well format, using CHIKV-nLuc. Three 384-well assay validation plates featuring alternating columns of DMSO vehicle (0.1%; max/maximum) or the broad-spectrum host-directed inhibitor cycloheximide in intermediate (0.5 × EC_50_ [0.1 µM; med/medium) or sterilizing (10 × EC_50_ [100 µM; min/minimum) concentrations. On each validation plate, control columns were arranged in a different order. (**C**) Performance parameters for all three plates and statistical assessments are shown. Signal-to-background (S/B), coefficients of variation for max, med, and min signals, and *Z*′ scores for each reference plate are shown. (**D**) Schematic of the HTS process. (**E**) Compounds were initially tested in a single concentration (5 µM), 384-well format. Primary hit analysis was performed using control-dependent (normalized percent inhibition) and control-independent (robust *Z*-score) methods. (**F**) Primary hits were tested in a 384-well dose-response format for primary target activity, toxicity, and activity against unrelated viruses to test for anti-CHIKV activity, toxicity, and promiscuity, respectively.

**Fig 2:**
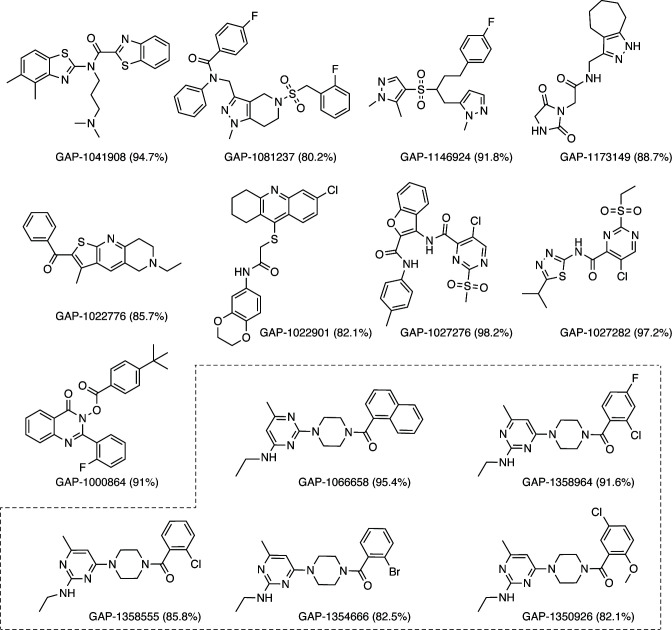
Primary screening hits. 14 compounds representing 10 unique scaffolds passed initial 384-well dose-response counterscreens. Normalized percent inhibition for each compound in our primary screen is shown in parentheses. These compounds were sourced from their respective vendors and selected for additional 96-well dose-response assays. Compounds belonging to the anti-CHIKV chemotype previously identified by Abdelnabi et al. (34) are outlined (dashed line).

**Fig 3:**
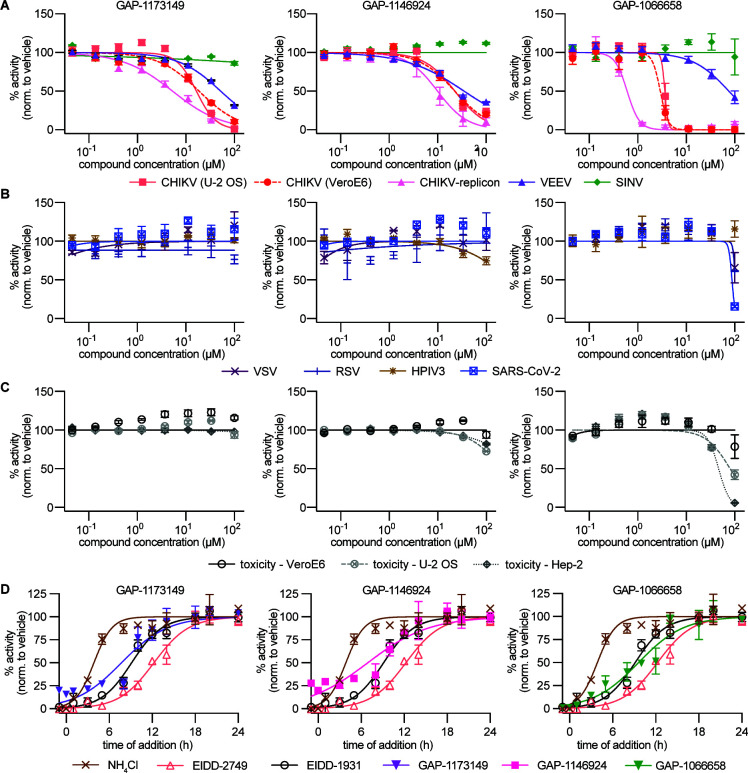
Validated hit scaffolds. (**A–C**) 96-well dose-response testing was performed on GAP-1173149 (left), GAP-1146924 (center), and GAP-1066658 (right). GAP-1173149 and GAP-1146924 displayed alphavirus-specific (**A**) activity with no measurable activity against unrelated viruses (**B**) and favorable toxicity profiles (**C**) (CC50 > 100 µM; 72-h incubation). Data are the mean ± standard error of the mean of three independent experiments. (**D**) Time-of-addition (ToA) profiles of GAP-1173149 (purple triangles, left), GAP-1146924 (magenta squares, center), GAP-1066658 (green triangles, right). EIDD-1931 (black open circles) and 4′-FlU (pink open triangles) were used as internal references for polymerase inhibitors. Ammonium chloride was used as a reference for entry inhibition. Time-of-addition studies were performed in U-2 OS cells. Data are the mean ± s.d. of three independent experiments.

We developed an HTS protocol using a recombinant CHIKV (181/25 strain) that expresses nanoluciferase (CHIKV-nLuc) (Fig. 1A). After validating this assay (Fig. 1B and C), we screened a 410,000-compound library (Fig. 1D and E). The HTS campaign performed well, with a mean *Z*′=0.5 and an average signal-to-background ratio of 32. By combining control-dependent methods (normalized percent inhibition, NPI) and control-independent methods (robust Z-score) (Fig. 1E), we identified 740 compounds for secondary orthogonal counter-screens in a 384-well format (Fig. 1F). The counter-screen criteria required EC_50_ <5 µM and CC_50_ >20 µM. Primary hit candidates were further processed *in silico* through PAINS filters (32) and aggregation predictions using Badapple2 (33) to identify compounds with potential structural liabilities (aggregators, chemically reactive structures), or known assay-interfering structures. Compounds showing activity against unrelated viral families, causing reporter interference, or exhibiting structural liabilities were excluded. From these 740 compounds, 14 hits, representing 10 distinct chemotypes, met all performance criteria in 384-well dose-response assays (Fig. 1F and Fig. 2). These hits were then procured for 96-well dose-response confirmation (Fig. 3A and B; Table 1). Although the potency of the compounds sourced from their respective vendor was slightly decreased compared to HTS library stocks used in 384-well counterscreens (below the initial HTS cutoff of EC_50_>5 µM), each confirmed hit displayed favorable bioactivity profiles (alphavirus-specific activity and CC_50_>100 µM). Therefore, these three chemotypes were selected for further investigation (Fig. 3A through D). However, one chemotype, represented by GAP-1066658, was previously identified as an inhibitor of the CHIKV capping machinery (34, 35). Although we excluded this chemotype from additional testing, it validated our anti-CHIKV HTS platform. The remaining two chemotypes, GAP-1146924 and GAP-1173149, were advanced for mechanistic investigations.

**TABLE 1:** Bioactivity profiles of primary hit candidates^*a*^

Compound ID	Antiviral potency (EC50 [µM])									Toxicity (CC50 [µM])	
	CHIKV (U-2 OS)	CHIKV (Vero)	CHKV replicon	VEEV	SINV	HPIV3	SARS-CoV-2	RSV	VSV	VeroE6	U-2 OS
GAP-1041908	13.3	22.2	32.1	18.8	18.9	41	28	n.d.^*b*^	32.7	34.9	31.4
GAP-1081237	9.1	10.5	12.4	10.0	65.7	64.9	>100	>10	65.2	>100	>100
GAP-1146924	24.1	19	9.2	35.2	>100	>100	>100	>10	>100	>100	>100
GAP-1173149	15.6	18.6	5.3	52.3	>100	>100	>100	>10	>100	>100	>100
GAP-1022776	9.1	7.7	n.d.	n.d.	>20	>20	>20	>10	>20	25.2	19.2
GAP-1022901	>10	3.76	n.d.	n.d.	>20	>20	>20	>10	>20	>20	>20
GAP-1027276	~10	~10	n.d.	n.d.	>20	>20	>20	>10	>20	>20	20
GAP-1027282	1.9	>10	n.d.	n.d.	>20	>20	>20	>10	>20	>20	16.7
GAP-1000864	~10	>10	n.d.	n.d.	>20	>20	>20	>10	>20	>20	>20
GAP-1066658	3.1	3.2	0.61	75.8	>100	~100	~100	>100	60.9	88.5	61.5

^*a*^ SINV, HPIV3, VSV, and VEEV potency was assessed on VeroE6 cells. RSV potency was assessed on Hep-2 cells. SARS-CoV-2 potency was assessed on VeroE6-TMPRSS2 cells.

^*b*^ n.d., not determined.

### Indication spectrum and mechanistic characterization

GAP-1146924 and GAP-1173149 both showed activity that appeared specific to alphaviruses, as neither compound inhibited human parainfluenza virus 3 (HPIV3), respiratory syncytial virus (RSV), SARS-CoV-2, or vesicular stomatitis virus (VSV), and neither displayed detectable toxicity at concentrations exceeding 100 µM (in VeroE6 and U-2 OS cell lines) (Fig. 3A through C; Table 1). Neither inhibited the alphavirus Sindbis virus (SINV), which is relatively distant from CHIKV. Time-of-addition studies using CHIKV-nLuc were then conducted to gain preliminary insight into which stage of viral replication is targeted by these compounds (Fig. 3D). Previously characterized CHIKV polymerase inhibitors 4′-FlU and EIDD-1931 (27, 29) served as polymerase-inhibition controls, while ammonium chloride treatment was used as an entry-inhibition reference (36). The ToA profiles of GAP-1146924 and GAP-1173149 were distinct from ammonium chloride and closely resembled those of 4′-FlU and EIDD-1931, suggesting these compounds act on a stage after viral entry, potentially targeting the viral nonstructural proteins involved in RNA synthesis (nsP1-4) or in polyprotein processing (nsP2) (Fig. 3D). To evaluate their potential as CHIKV replicase inhibitors, we initially tested GAP-1146924 and GAP-1173149 in dose-response replicon assays (Table 1). Both compounds exhibited low micromolar potency (EC_50_ of 9.2 µM and 5.3 µM for GAP-1146924 and GAP-1173149, respectively) in replicon assays, providing additional support for the hypothesis that they targeted the viral nonstructural proteins (Fig. 3A; Table 1).

**Fig 4:**
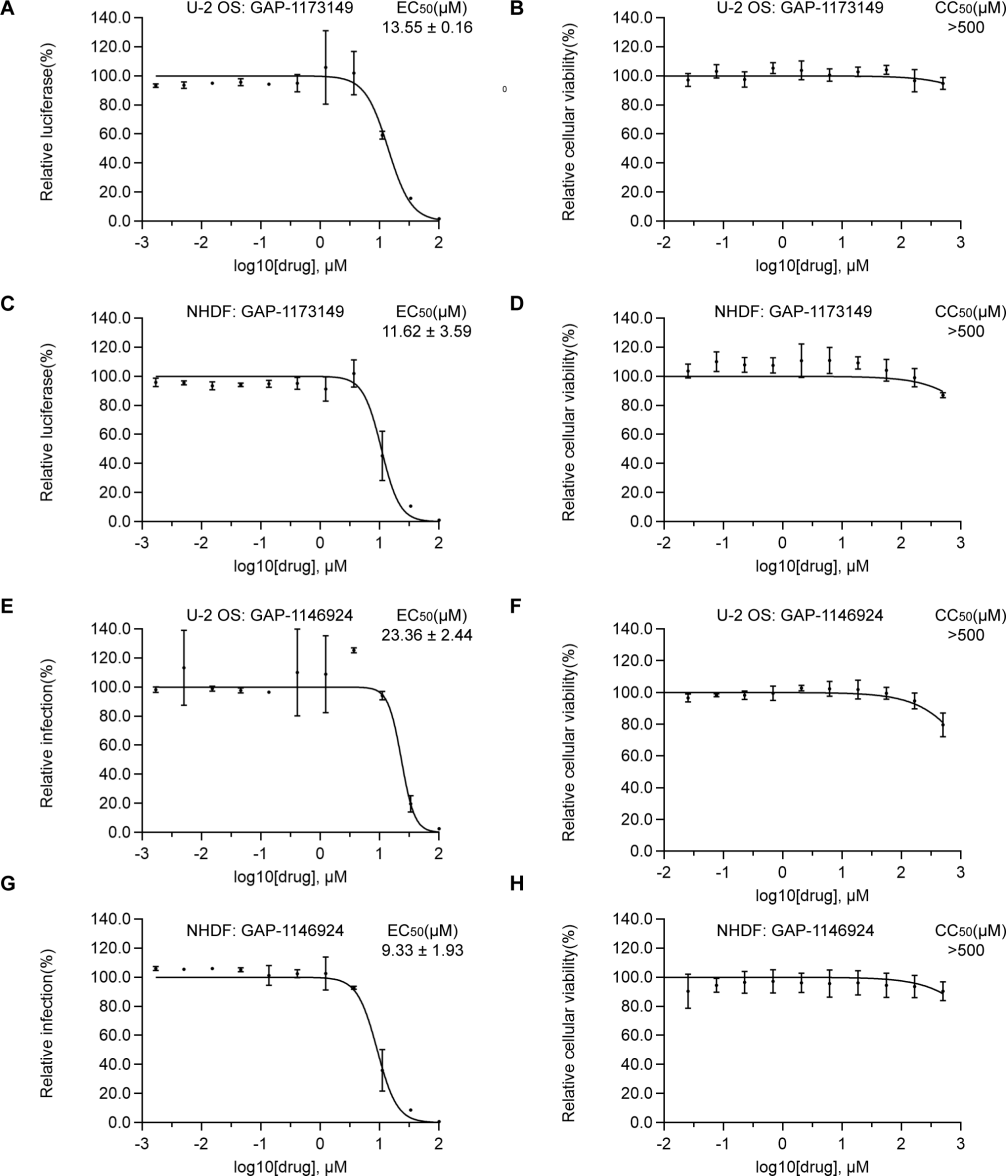
Dose-response studies of GAP-1173149 and GAP-1146924 in CHIKV-infected NHDF cells. (**A, C, E and G**) Effect of GAP-1173149 (**A and C**) or GAP-1146924 (**E and G**) on CHIKV-nLuc. U-2 OS (**A and E**) or NHDF (**C and G**) cells were infected with CHIKV-nLuc at a multiplicity of infection (MOI) of 0.1 focus-forming unit (FFU)/cell for 1 h and then treated with the indicated concentrations of GAP-1173149 or GAP-1146924. Nanoluciferase activity was measured at 24 hpi and normalized to that of control cells. (**B, D, F and H**) U-2 OS (**B and F**) or NHDF (**D and H**) cells were incubated with the indicated concentrations of compound for 24 h. Cell viability was assessed using PrestoBlue and normalized to control DMSO-treated cells. Symbols in all panels represent the mean ± s.d. of three independent experiments.

Next, we assessed the antiviral efficacy of GAP-1173149 and GAP-1146924 against CHIKV infection of primary normal human dermal fibroblast (NHDF) cells, as dermal fibroblasts are a key initial target of CHIKV infection (5, 37). GAP-1173149 or GAP-1146924 inhibited nLuc production with an EC_50_ of 11.62 µM or 9.33 µM, respectively (Fig. 4A and C), similar to the EC_50_ observed in the U-2 OS cells used for the HTS. Analysis of GAP-1173149 and GAP-1146924 cytotoxicity in NHDF cells showed that the CC_50_ for both compounds was greater than 500 µM (Fig. 4B and D), resulting in a high selectivity index (SI = CC_50_/EC_50_, >43 or >54, respectively).

### Antiviral breadth of GAP-1173149 or GAP-1146924 against alphaviruses

**Fig 5:**
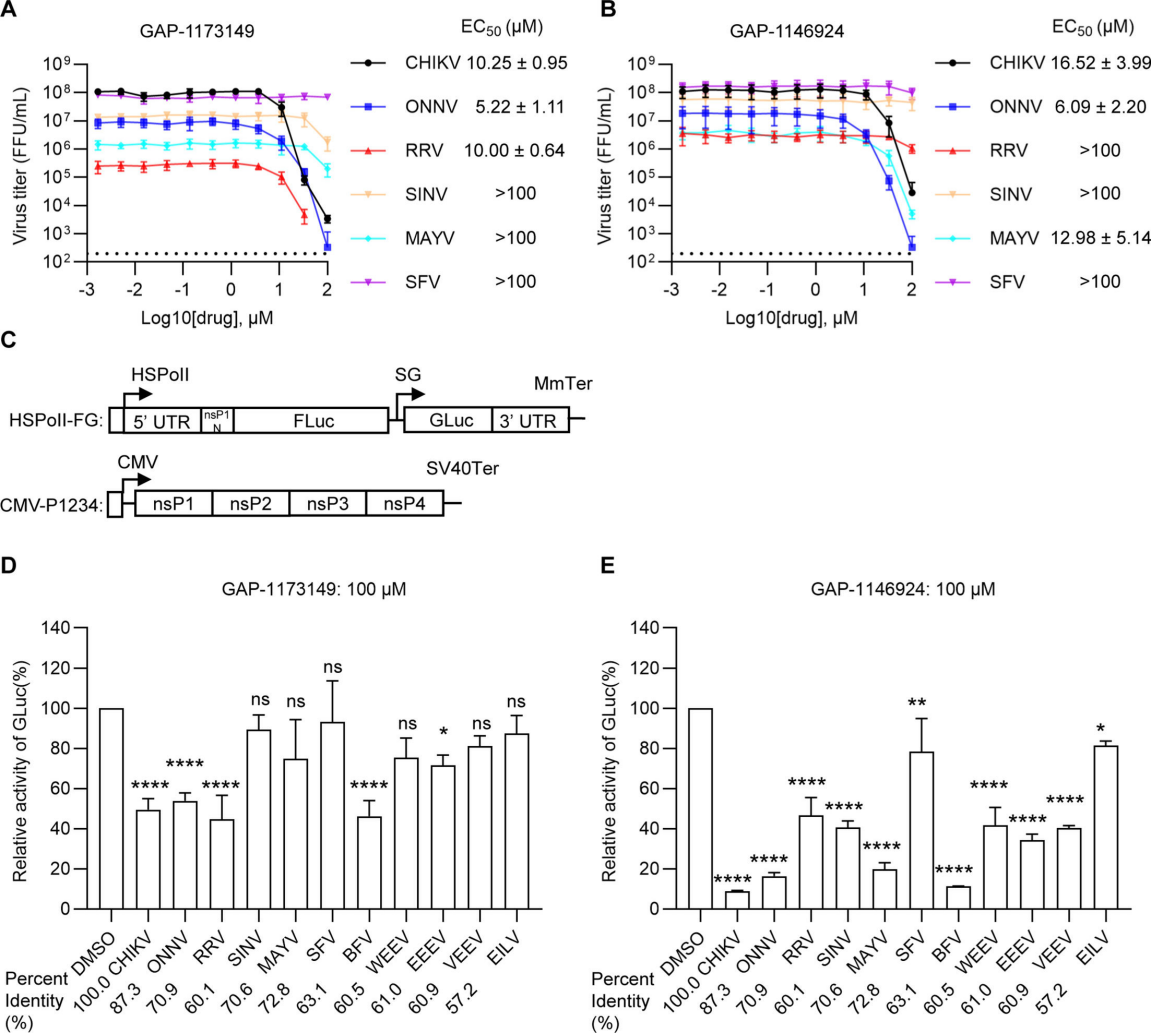
Antiviral breadth of GAP-1173149 or GAP-1146924 against alphaviruses. (**A and B**) Effect of GAP-1173149 (**A**) or GAP-1146924 (**B**) on the production of various alphaviruses. U-2 OS cells were inoculated with various alphaviruses (MOI 0.1 FFU/cell) and treated with the indicated concentrations of compound. Culture supernatants were collected at 24 hpi, and the results of this multi-cycle infection were titered by FFA on U-2 OS cells. Data are the mean ± s.d. of three independent experiments. (**C**) Schematic of expression plasmids for the alphavirus *trans*-replicase assay, showing template RNA (top) and viral replicase protein expression constructs (CMV-P1234). Annotations indicate the following: HSPolI, a truncated human RNA polymerase I promoter (nucleotides −211 to −1); MmTer, the mouse RNA polymerase I terminator; CMV, the human cytomegalovirus immediate early promoter; HSV TK Ter,the herpes simplex virus thymidine kinase terminator. (**D and E**) U-2 OS cells were transfected with alphavirus *trans*-replicase constructs for 4 h and treated with indicated concentrations of GAP-1173149 (**D**) or GAP-1146924 (**E**) for 20 h. GLuc activity was determined and normalized to that of DMSO-treated controls. Data are the mean ± s.d. of three independent experiments. Statistical significance was determined by one-way ANOVA with Dunnett’s test compared to the DMSO control. *P < 0.05, **P < 0.01, ****P < 0.0001. The percent identity of each virus to CHIKV is shown under the D and E panels and was determined by multiple sequence alignment of alphavirus nonstructural proteins (nsP1–nsP4). Swiss-Prot accession numbers: CHIKV: A4L7I2, ONNV: P13886, MAYV: Q8QZ73, SFV: P08411, RRV:P13887, BFV: P87515, SINV: P03317, WEEV: P13896, VEEV: P36328, EEEV: Q306W6. GenBank accession number of EILV: BFZ80397.

To evaluate the broad-spectrum antiviral potential of GAP-1173149 and GAP-1146924 across the alphavirus genus, we tested their inhibitory activity against a panel of re-emerging pathogens (ONNV, RRV, and MAYV), as well as the less pathogenic SINV and Semliki Forest virus (SFV). U-2 OS cells were inoculated with the indicated alphavirus at an MOI of 0.1 FFUs per cell for 1 h. Cells were then treated with varying concentrations of GAP-1173149 or GAP-1146924, and supernatants were collected at 24 hpi and titrated to determine the effect of the compounds on multi-cycle infection. GAP-1173149 effectively suppressed the replication of CHIKV, ONNV, and RRV, showing comparable EC_50_ values for all three viruses (Fig. 5A). In contrast, GAP-1146924 demonstrated inhibitory activity against CHIKV, ONNV, and MAYV but not RRV (Fig. 5B). To further investigate the inhibitory effects on viral RNA replication across various alphaviruses, especially high-risk encephalitic alphaviruses (belonging to biosafety level 3), we employed a trans-replicase system in which expression of the P1234 polyprotein drives replication of a reporter RNA template (Fig. 5C). In this system, 100 µM GAP-1173149 effectively inhibited RNA replication of EEEV and Barmah Forest virus (BFV). It also suppressed replication of CHIKV, ONNV, and RRV, but showed no significant effect on MAYV, SINV, or SFV (Fig. 5D), consistent with the virus production results shown in Fig. 5A. At the single 100 µM concentration tested, GAP-1146924 showed some inhibition of RNA replication across all the tested alphavirus constructs, including WEEV, EEEV, VEEV, and Eilat virus (EILV), an insect-specific alphavirus (Fig. 5E).

We note that in the trans-replicase assay, unlike during virus infection, the nsPs need to find and interact in *trans* with mRNA molecules rather than react in *cis* with the viral genome. The in *trans* process is more sensitive to inhibition and can be affected by the role of nsP4 in template selection and engagement (38). This may explain why we observed some GAP-1146924 inhibition of RRV, SINV, and SFV in the trans-replicase assay but not in virus production.

We also note that the initial counter-screen observed some inhibition of VEEV-nLuc when GAP-1173149 was tested in Vero E6 cells (Table 1), while the trans-replicase assays did not show significant VEEV inhibition in human U-2 OS cells. The VEEV-nLuc used in the counterscreen dose-response assays encoded a PEST degron sequence fused to the C-terminus of nLuc (31). The increased turnover rate and lack of accumulated signal for nLuc-PEST may account for the differences in inhibition observed for these assays compared to the trans-replicase assay, which uses a standard GLuc reporter.

### GAP-1173149 and GAP-1146924 select for mutations in the CHIKV RdRp

**Fig 6:**
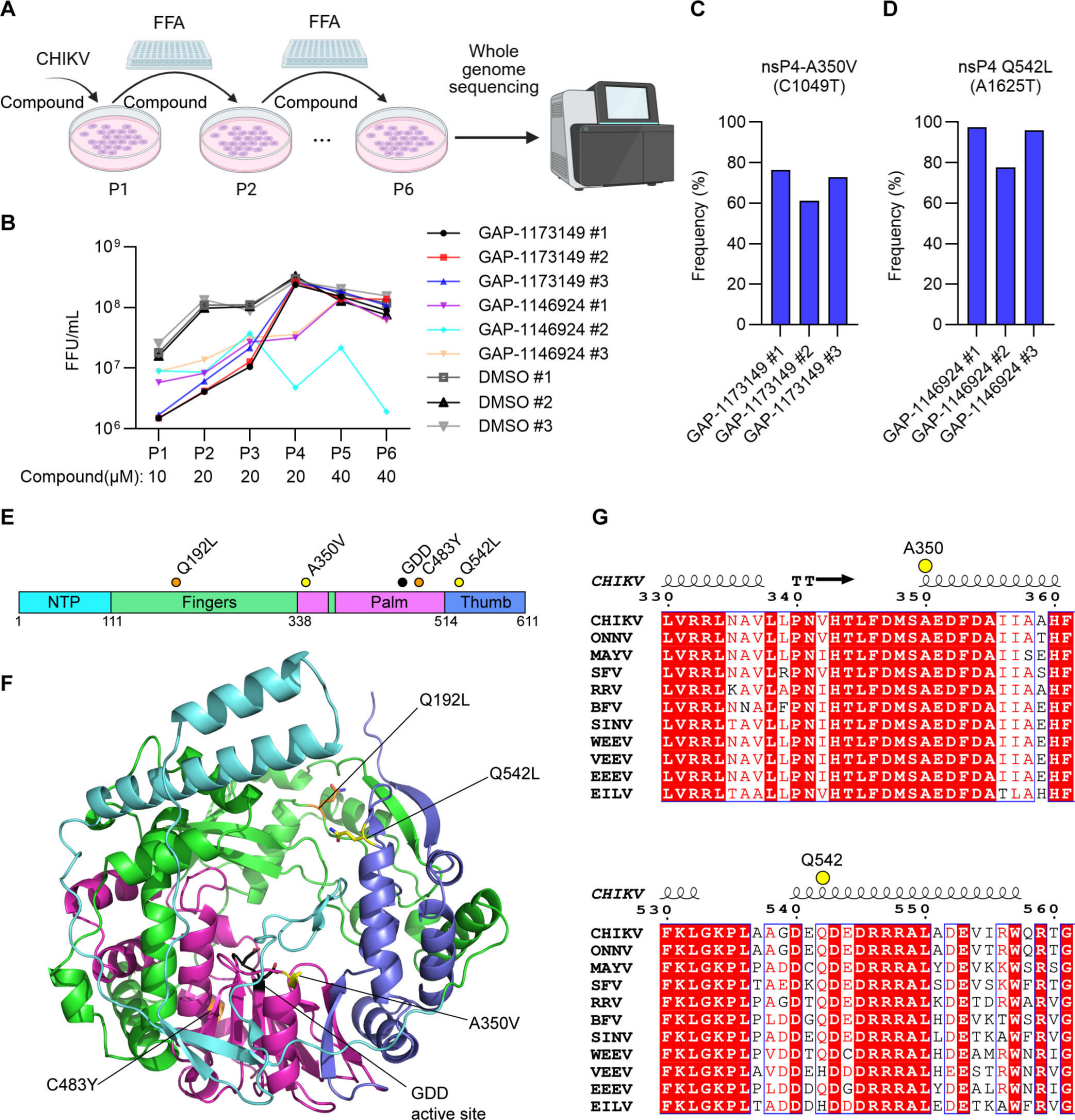
GAP-1173149 or GAP-1146924 selects for mutations in the CHIKV RdRp. (**A**) Schematic overview of the *in vitro* selection workflow for CHIKV under compound pressure. Figure created in BioRender. Yin, P. (2025) https://BioRender.com/ifpcoki. (**B**) Viral replication dynamics across three independent CHIKV lineages subjected to serial passaging in U-2 OS cells under indicated concentrations of GAP-1173149, GAP-1146924, or DMSO control. Viral titers were quantified by FFA after each passage. (**C**) Mutation frequencies in viral populations from three GAP-1173149-treated lineages after passage 6, as determined by WGS. This mutation was not observed in virus populations treated with GAP-1146924 or DMSO. (**D**) Mutation frequencies in three GAP-1146924-treated viral populations after passage 6, as determined by WGS. These mutations were not detected in GAP-1173149 or DMSO-treated virus populations. (**E**) Schematic of the CHIKV nsP4 polymerase. Locations of resistance mutations identified with GAP-1173149 (A350V) and GAP-1146924 (Q542L) are shown as yellow circles. Resistance mutations previously identified against 4′-FlU are shown as orange circles. The GDD active site is denoted with black circles. (**F**) Locations of antiviral resistance mutations in a 3D-model of the CHIKV nsP4 polymerase. Stick representations are used to indicate the following residues: Mutations identified with GAP-1173149 (A350V) and GAP-1146924 (Q542L) are shown in yellow. Resistance mutations against 4′-FlU are shown in orange. The GDD active site is shown in black. The NTP, fingers, palm, and thumb domains are colored cyan, green, magenta, and blue, respectively (**E and F**). (**G**) Alignment of alphavirus nsP4 sequences around residues A350 or Q542. These residues are indicated by yellow dots. TT stands for tight turn. Swiss-Prot accession numbers: CHIKV: A4L7I2, ONNV: P13886, MAYV: Q8QZ73, SFV: P08411, RRV: P13887, BFV: P87515, SINV: P03317, WEEV: P13896, VEEV: P36328, EEEV: Q306W6. GenBank accession number of EILV: BFZ80397.

To define the resistance profile and identify the viral targets of GAP-1173149 and GAP-1146924, we serially passaged CHIKV in U-2 OS cells in the presence of low concentrations of compound (Fig. 6A and B). Cells were inoculated at an MOI of 1 FFU/cell and cultured for 16 h in GAP-1173149, GAP-1146924, or DMSO (three independent lineages for each condition). Viral titers were quantified after the first passage (P1), and cells were subsequently infected using the same MOI for the remaining rounds of selection. In GAP-1173149-selected samples, virus titers at P1 were reduced by 1 log compared with DMSO-treated controls. All three GAP-1173149 lineages exhibited resistance to P4. To further select for resistance, two additional passages were performed at an increased concentration of GAP-1173149, generating P6 virus stocks (concentrations as specified in Fig. 6B). For GAP-1146924, two out of three lineages exhibited resistance to the higher concentration of drug by P5 and maintained resistance by P6. Lineage #2 of GAP-1146924 displayed some resistance by P3 but was inhibited when exposed to increased drug concentration in P4-6. RNA was extracted from all the P6 stocks, reverse transcribed, and subjected to whole-genome sequencing (WGS) using Illumina deep sequencing technology.

Mutations in nsP4 were detected at high frequencies in P6 viral stocks selected with GAP-1173149 or GAP-1146924 (Table 2). All three GAP-1173149 stocks harbored an nsP4-A350V mutation at frequencies ranging from 61% to 76.3% (Fig. 6C). Lineages 1 and 3 of GAP-1146924 P6 stocks contained an nsP2-Q542L mutation at high frequencies (97.5% or 95.9%) (Fig. 6D). Lineage 2 contained both nsP4-Q542L (77.5%) and nsP4-Q192P (23.6%), with a combined allele frequency about 100% (Table 2). The fact that these allele frequencies (nsP4-Q542L and Q192P) add up to ~100% strongly suggests that these two mutations are unlinked, although given their distance apart (1,050 nt) vs our normal sequence fragments (400–500 bp), we cannot definitively conclude this. No mutations were identified at frequencies exceeding 10% in the DMSO-treated control lineages 1–3. The nsP4-A350V mutation is located within the palm domain and the nsP4-Q542L is within the thumb domain of nsP4 (Fig. 6E). These mutations are distinct from the previously identified Q192L and C483Y mutations that confer resistance to the nucleoside analog 4′-FlU (Fig. 6E). In the CHIKV nsP4 polymerase structure, residue A350 is positioned adjacent to the conserved GDD active site (Fig. 6F). Interestingly, in the nsP4 structure, Q542 is in relatively close proximity to Q192, although these two residues are located in different domains. A350 is highly conserved across the alphavirus genus, whereas Q542 corresponds to a histidine (H) in VEEV and EILV (Fig. 6G).

**TABLE 2:** Mutations with frequency >20% in selected CHIKV lineages

Sample	Frequency (%)	Change type	AA change	Nucleotide change
GAP-1173149 lineage #1	73.77	Synonymous	NA^*a*^	nsP3: A372C
	76.32	Nonsynonymous	nsP4: A350V	nsP4: C1049T
GAP-1173149 lineage #2	59.61	Synonymous	NA	nsP3: G1422T
	61.05	Nonsynonymous	nsP4: A350V	nsP4: C1049T
GAP-1173149 lineage #3	72.91	Nonsynonymous	nsP4: A350V	nsP4: C1049T
GAP-1146924 lineage #1	97.51	Nonsynonymous	nsP4: Q542L	nsP4: A1625T
GAP-1146924 lineage #2	23.56	Nonsynonymous	nsP4: Q192P	nsP4: A575C
	77.51	Nonsynonymous	nsP4: Q542L	nsP4: A1625T
GAP-1146924 lineage #3	95.9	Nonsynonymous	nsP4: Q542L	nsP4: A1625T

^*a*^ NA indicates not applicable.

### The nsP4 mutations specifically confer resistance to GAP-1173149 and GAP-1146924

**Fig 7:**
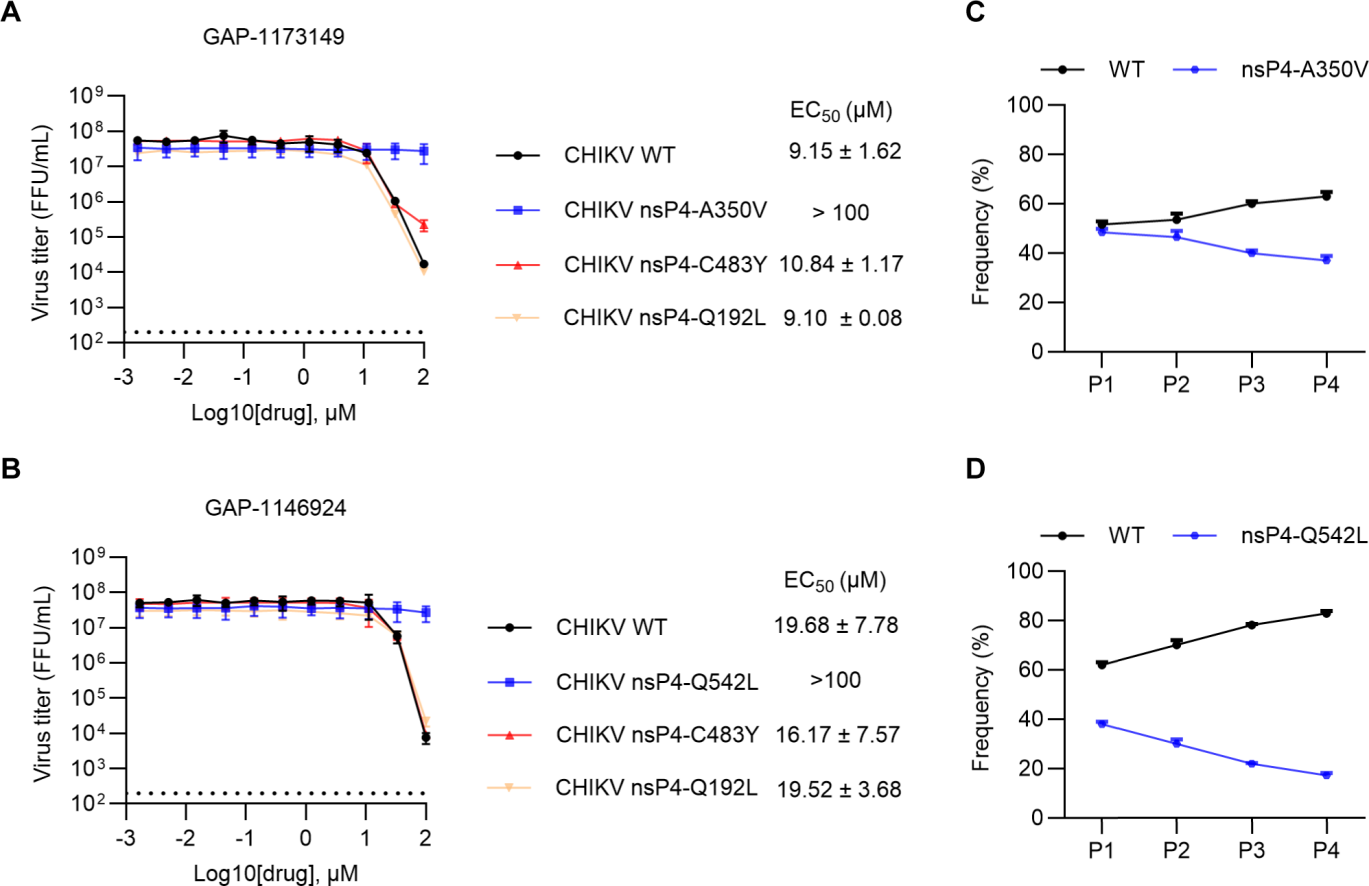
NsP4-A350V or nsP4-Q542L confers resistance to GAP-1173149 or GAP-1146924, respectively. (**A and B**) Targeted mutations were introduced into the CHIKV 181/25 genome, including nsP4-Q192L and nsP4-C483Y, which confer resistance to 4′-FlU, and the resulting mutant virus stocks were assessed for sensitivity to GAP-1173149 (**A**) or GAP-1146924 (**B**). Viral production was measured following treatment with the indicated concentrations, as described in Fig. 5A. Data are the mean ± s.d. from three independent experiments. (**C and D**) Competition assays assessing relative viral fitness in U-2 OS cells. Cells were inoculated at an MOI of 0.1 FFU/cell with 1:1 mixtures (P1) of the indicated 181/25 WT and mutant viruses and incubated for 24 h. Viruses were serially passaged three times (P2–P4) at an MOI of 0.1 FFU/cell. Viral frequencies in the P1–P4 stocks were determined by WGS. Values represent the mean of three technical replicates from a single experiment.

To elucidate the role of nsP4-A350V and nsP4-Q542L, we introduced these mutations individually into the CHIKV 181/25 infectious clone, generated virus stocks, and assessed their susceptibility to GAP-1173149 or GAP-1146924. The nsP4-A350V substitution exhibited resistance to GAP-1173149, maintaining viral replication even at a concentration of 100 µM (Fig. 7A). Similarly, the nsP4-Q542L substitution showed resistance to 100 µM GAP-1146924 (Fig. 7B). Thus, each of these mutations results in appreciable CHIKV resistance. CHIKV carrying the Q192L or C483Y mutations that confer resistance to 4′-FlU (30) were not resistant to either GAP-1173149 or GAP-1146924, confirming lack of cross-resistance (Fig. 7A and B). In contrast to 4′-FlU, which functions as a nucleoside analog and viral RNA chain terminator, these findings suggest that GAP-1173149 and GAP-1146924 inhibit nsP4 by distinct mechanisms. This is in keeping with the structures of these compounds, which do not suggest that either could act as a nucleoside analog.

We performed fitness tests of the mutants by mixing either the nsP4-A350V or nsP4-Q542L viruses with the WT virus at a 1:1 ratio (P1), inoculating the mixtures onto U-2 OS cells, and serial passaging the progeny virus three times. The frequency of WT and mutant was determined for each passage by WGS (Fig. 7C and D). The results showed that in each case the WT virus out-competed the mutant, indicating that resistance to either drug resulted in decreased fitness.

**Fig 8:**
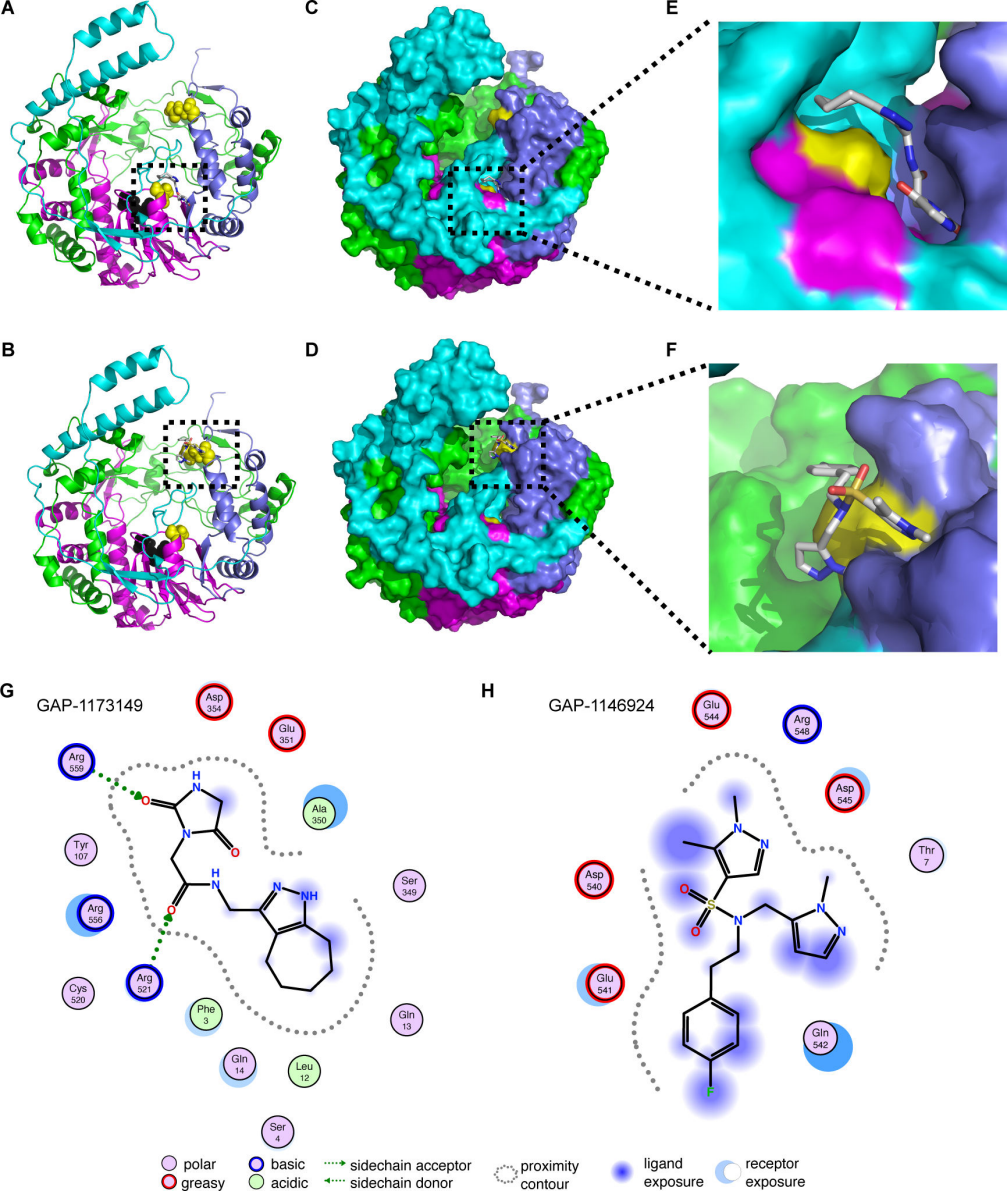
*In silico* docking. (**A and B**) Location of the top-scoring docking poses for GAP-1173149 (**A**) and GAP-1146924 (**B**) is shown (dashed outline). (**C and D**) Location of the docking poses within a surface representation of the ONNV nsP4 polymerase (PDB ID: 7Y38). (**E and F**) Close-up of the *in silico* docking poses for GAP-1173149 (**E**) and GAP-1146924 (**F**). Resistance mutations are shown as yellow spheres (A350V for **A, C, and E**; Q542L for **B, D, and F**). The GDD active site is shown as black spheres (**A and B**). The NTP, fingers, palm, and thumb domains are colored cyan, green, magenta, and blue, respectively. (**G**) GAP-1173149 docks within a pocket adjacent to the GDD active site. For GAP-1173149, predicted are hydrogen bond interactions between E553 and the amide group of the hydantoin ring of GAP-1173149. (**H**) For GAP-1146924, the compound docks opposite the GDD active site with large portions of the molecule being solvent-exposed. Hydrogen bonds are predicted to form between R593 and an oxygen of the sulfonyl group.

Based on the locations of mutations identified in the susceptibility profiling studies, we performed *in silico* docking using the structure of the ONNV nsP4 polymerase (PDB 7Y38) (39). Residue A350 was selected as the docking target for GAP-1173149, and Q542 for GAP-1146924. The predicted top-scoring docking poses for each compound are shown in Fig. 8 (Table 3). For GAP-1173149, the top-scoring predicted docking pose places the compound near the GDD active site, in a small pocket located in immediate proximity to residue A350. In contrast, the top-scoring prediction for GAP-1146924 positions the compound on the opposite side of the GDD active site, near the previously reported Q192L mutation that confers 4′-FlU resistance (30). Unlike the GAP-1173149 docking pose, a large portion of the pose for GAP-1146924 is solvent-exposed (Fig. 8H). The docking poses presented here are informed by resistance mapping. In the absence of direct biochemical binding data and definitive microdomain mapping, compound placement was guided by the spatial position of the resistance mutation identified for each antiviral. While these models will require biochemical and structural validation, the convergence of independent resistance lineages on discrete regions of nsP4 provides strong genetic evidence supporting this polymerase as the molecular target of inhibition. Notably, resistance mutations localize to regions of nsP4 that are not implicated in interactions with other components of the viral replication machinery (nsP1, nsP2, and nsP3), arguing against disruption of protein-protein interfaces and instead supporting direct inhibition of polymerase function (39). The resistance profiles for both compounds are consistent with established paradigms for allosteric small-molecule polymerase inhibitors, in which substitutions arise proximal to allosteric binding pockets and alter local structural environments that govern compound engagement (40–46). Although distal or long-range structural effects cannot be excluded, our data most support a model in which resistance emerges from perturbations within or adjacent to the compound-binding sites. As additional structural and biophysical validation studies are completed, these models will provide a rational framework for iterative structure-guided optimization.

**TABLE 3:** Summary of *in silico* docking scores^*a*^

Compound	Target	S	rmsd
GAP-1146924	ONNV nsP4 Q542	−5.1070285	1.4327091
GAP-1173149	ONNV nsP4 A350	−6.4510121	0.9384039

^*a*^ S denotes free energy scores (kcal/mol) for final docking poses. rmsd quantifies the structural difference (Å) of the ligand pose before and after refinement.

## DISCUSSION

Targeting the CHIKV RdRp with small-molecule inhibitors represents a promising antiviral strategy. CHIKV nsP4 is essential for viral genome replication and transcription, making it an attractive target. By inhibiting the activity of the viral polymerase, replication can be halted early in the viral life cycle, shutting down viral RNA synthesis, replication, and, consequently, disease progression. In addition, the polymerase tends to be relatively well conserved among related viral species, allowing for cross-reactivity against other alphaviruses, thus enabling broad-spectrum coverage. Previous experience with other RNA virus families has demonstrated that direct-acting polymerase inhibitors can be highly effective while retaining acceptable safety profiles. For instance, polymerase inhibitors have significantly improved clinical outcomes for HIV, hepatitis C virus, and other RNA viruses (47–52). Although a CHIKV vaccine is available, small molecule inhibitors can provide an additional measure to supplement protection in areas where vaccination coverage is low. This is particularly important in outbreak situations, when immediate therapeutic intervention is needed to control the spread of disease.

Here, we have identified and validated two hit chemotypes, GAP-1173149 and GAP-1146924, that inhibit CHIKV in live-virus assays in both immortalized human cell lines and primary human cells. Both hit candidates exhibited broad activity against multiple species within the *Alphavirus* genus. Initial mechanistic characterization suggested that the viral replication machinery was the target of inhibition. Both GAP-1173149 and GAP-1146924 inhibited RNA replication in trans-replicase assays of CHIKV, ONNV, RRV, BFV, and EEV. GAP-1146924 also inhibited RNA replication in trans-replicase assays of the encephalitic alphaviruses WEEV and VEEV. To map the viral protein target and assess resistance barriers, we passaged CHIKV in the presence of increasing concentrations of either GAP-1173149 or GAP-1146924. We identified mutations within the CHIKV nsP4 protein and confirmed that they confer reduced sensitivity to the inhibitor. Competition studies between mutant and WT showed that the mutations decreased virus fitness in cell culture. Together, our data suggest that these two compounds target distinct sites within the CHIKV nsP4 polymerase. Their chemotypes and resistance profiles suggest that they may act as allosteric inhibitors of nsP4.

A primary concern for the clinical performance of small-molecule antivirals is the emergence of drug resistance. However, characterizing resistance mechanisms early in drug development establishes a robust foundation to inform medicinal chemistry, with the objective to potentially boost both potency and barriers to resistance. By systematically characterizing polymerase-drug interactions, lead scaffolds could potentially be refined proactively to address viral escape mutants while preserving antiviral activity. In addition, combinatorial use of inhibitors can greatly decrease the likelihood of the generation of resistance mutants within the time frame of acute CHIKV infection.

Although GAP-1173149 and GAP-1146924 exhibit broad activity across multiple alphaviruses, neither compound demonstrates pan-alphavirus inhibition. The variability in antiviral activity across distinct alphavirus species is consistent with the established mechanism of action of allosteric polymerase inhibitors (40–46). In contrast to conserved catalytic domains targeted by nucleoside analogs, allosteric binding sites exhibit substantially lower sequence and structural conservation. As a result, small-molecule engagement of these sites is inherently sensitive to subtle changes in the local structural and physicochemical environment surrounding the binding pocket. Even within alphaviruses, nsP4 proteins share only moderate sequence identity, and relatively minor amino acid differences within or proximal to allosteric pockets can have drastic effects on inhibitor binding, resulting in a loss of activity. Consistent with our data, CHIKV nsP4 is phylogenetically more similar to ONNV than to SINV nsP4 (38), providing additional support for data showing similar potency between ONNV and CHIKV, but not SINV (Fig. 5). If the compound relies on an allosteric pocket that is present in CHIKV and ONNV nsP4 but absent, occluded, or structurally collapsed in SINV nsP4, antiviral activity would be expected to diminish or be lost entirely. Although the docking poses presented here represent hypothetical models of compound engagement, comparative structural analysis between ONNV (PDB ID: 7Y38) and SINV (PDB ID: 7VW5) nsP4 structures reveals discernible differences within regions proximal to the proposed binding microdomains (39, 53). Structural variation surrounding residues 551–556 (GAP-1173149) and 537–539 (GAP-1146924) provides a plausible structural basis for the diminished SINV susceptibility. Moreover, alphavirus polymerases undergo substantial conformational transitions during initiation and elongation (54). CHIKV and ONNV nsP4 may readily adopt conformations that expose the inhibitor binding regions, whereas SINV nsP4 either disfavors or only transiently adopts this state, thereby limiting productive inhibitor engagement.

In conclusion, we have identified much-needed novel small-molecule inhibitors of CHIKV RNA synthesis. Our data suggest that these compounds target previously unrecognized druggable sites within the alphavirus RdRp. These two chemotypes represent a starting point for additional development to increase potency and broad-spectrum activity. Continued efforts to identify and optimize these small-molecule hit candidates will advance not only our understanding of viral replication mechanisms but also the development of novel treatment options for CHIKV and related emerging alphaviruses.

## MATERIALS AND METHODS

### Cells

Human osteosarcoma (U-2 OS; ATCC HTB-96) cells were maintained at 37°C and 5% CO2 in McCoy’s media supplemented with 10% fetal bovine serum (FBS). Human carcinoma (HEp-2; ATCC CCL-23), baby hamster kidney cells (BHK-21; ATCC CCL-10), African green monkey kidney epithelial cells (VeroE6; CRL-1586; ATCC), and VeroE6 (CRL-1586; ATCC) stably expressing human TMPRSS2 (VeroE6-TMPRSS2; BBS Bioscience 78,081) were cultured in Dulbecco’s modified Eagle’s medium (DMEM) supplemented with 7.5% FBS. Normal human dermal fibroblasts (NHDF; ATCC PCS-201-012) were cultured in DMEM containing 10% FBS. HEp-2 cells are listed in the International Cell Line Authentication Committee database of commonly misidentified cell lines. The use of these cells is necessary due to their permissiveness for RSV. All immortalized cell lines are routinely checked for microbial contamination and Mycoplasma. All cells were maintained at 37 °C and 5% CO_2_.

### Molecular biology

VEEV-TC83-nLuc was derived from VEEV-TC83 by adding a NanoLuc reporter cassette under the control of the subgenomic promoter, followed by an additional subgenomic promoter to express structural proteins. VEEV-nLuc stocks were authenticated by whole-genome sequencing. The infectious cDNA clone of the CHIKV-nLuc reporter virus (181/25 strain) was generated as previously described (29). To introduce specific mutations into the infectious cDNA clones of CHIKV, site-directed mutagenesis was performed using Gibson assembly. DNA fragments containing the nsP4-A350V mutation were generated by polymerase chain reaction (PCR) amplification with the following primers: BSAB1-F: CCAAATCACCGACGAGTATGATGCATATCTAGACATGGTG, nsP4-A350V-F: GCATACACTATTTGACATGTCTGTCGAGGACTTCGATGCCATTATAG, nsP4-A350V-R: CTATAATGGCATCGAAGTCCTCGACAGACATGTCAAATAGTGTATGC, and Swa1-R2: GCTAACGGTTTGCCCAATTTAAATAACCTTTTTAGCGGG. The resulting PCR products were assembled into linearized CHIKV infectious clones previously digested with BsaBI and SwaI, using the Gibson Assembly Master Mix (New England Biolabs, #E2611) according to the manufacturer’s protocol. The DNA fragments containing nsP4-Q542L mutation were generated using the following primers (5′→3′): NSP4-Q542L-Swa1-F: CCCGCTAAAAAGGTTATTTAAATTGGGCAAACCGTTAGCGGCAGGTGACGAACTA GATGA and SacI-R: GGTGAACCGGCCTCCTGAGTACTGTACTGCTCCGTGGTGCC. The PCR products were assembled into linearized CHIKV infectious plasmids previously digested with SwaI and SacI, using Gibson assembly as described above.

### Viruses

VEEV-TC83-nLuc virus encoding nLuc with a C-terminal PEST degron (31) was rescued by transfecting (Lipofectamine MessengerMAX Transfection; ThermoFisher Scientific) BHK-21 cells with *in vitro* transcribed RNA (mMESSAGE mMACHINE SP6, ThermoFisher Scientific) in accordance with the manufacturer’s protocol. The virus was harvested after 24 h, and titers were determined using TCID_50_ titration. Counterscreens to determine target specificity and identify potential host-directed compounds included HPIV3 nLuc, VSV-nLuc, and RSV-A2 L19F-Firefly. HPIV3 nLuc (44), VSV-nLuc (44), and RSV-A2 L19F-Firefly (55) were amplified on VeroE6 (HPIV3, VSV) and HEp-2 (RSV) cells (MOI = 0.01 TCID50 units per cell). HPIV3, VSV, and RSV stocks were purified on a 60% sucrose cushion. The identity of rescued viruses was confirmed through whole-genome sequencing. Titers of HPIV3, VSV, or RSV virus stocks were determined by TCID50 titration on Vero-E6 (HPIV3, VSV) or HEp-2 (RSV) cells, respectively. SARS-CoV-2 nLuc stocks were propagated on VeroE6-TMPRSS2 cells and titered by TCID50 assay on Vero-E6-TMPRSS2 cells.

Virus stocks used in the *in vitro* assays were generated from the following infectious cDNA clones: CHIKV 181/25 (pSinRep5-181/25ic [56], kindly provided by Dr. Terence S. Dermody), the CHIKV 181/25 mutants described above, MAYV (MAYV-CH-mKATE), RRV (RRV-T48-ZsGreen), and ONNV (ONNV-ZsGreen) (29), SFV (pSP6-SFV4) (57), SINV-GLuc (pToto1101-2SG-GLuc, from Andres Merits), and SINV (dsTE12Q [58], kindly provided by Dr. Beth Levine). Infectious cDNA clones were linearized with the appropriate restriction enzymes, purified, and subsequently used as templates for *in vitro* transcription. Capped RNA transcripts were synthesized using the SP6 mMessage mMachine kit (Life Technologies) in accordance with the manufacturer’s instructions. The resulting RNA was electroporated into BHK-21 cells to initiate virus recovery. Electroporated cells were incubated at 37°C for 24 hours. Virus-containing supernatants were then harvested and clarified by centrifugation at 3,000 rpm for 20 minutes at 4°C using a Thermo Scientific TX-1000 rotor. Viral titers of the resulting stocks were determined by focus-forming assay (FFA) in U-2 OS cells.

### HTS and hit identification

A total of 410,000 compounds (representing compounds present in the Chembridge Diverset and ChemDiv Representative Diversity open discovery libraries) were screened for inhibitory activity against CHIKV-nLuc (MOI = 0.3 TCID_50_ units per cell). HTS was carried out similarly to previous reports (46, 44) with a compound concentration of 5 µM and automated reading of plates 20 h after infection. Raw data were imported into the Chemical and Biological Information System (ChemInnovation Software, Inc.; CBIS) IT environment and analyzed as described (46, 44). Hit candidates were defined as compounds with anti-CHIKV activity of >5.3  ×  s.d. in control-dependent normalized percent inhibition and >1.45  ×  s.d. in control-independent mean robust *Z* score calculation.

### Counterscreens

For counterscreens, compound was added in threefold dilutions (20–0.009 μM or 100–0.046 µM) to 96-well plates seeded with U-2 OS, Hep2, or Vero-E6 cells (1.1 × 10^4^ per well), followed by infection with CHIKV-nLuc, VEEV-nLuc, HPIV3-nLuc, SARS-CoV-2-nLuc, VSV-nLuc, SINV-GLuc, or RSV A2-L19F reporter viruses (MOI = 0.2 TCID50 units per cell) and automated plate reading 24–36 h after infection. Cytotoxic concentrations were determined in identical serial dilution plates after exposure of uninfected cells for 72 h to the compounds. Cytotoxicity was assessed by the addition of PrestoBlue substrate (Invitrogen) to quantify cell metabolic activity, as described (44, 46). Four-parameter variable slope regression modeling was used to determine EC_50_ and CC_50_ concentrations. To determine potential structural liabilities (known aggregators, chemical reactivity (i.e., the presence of Michael acceptors, covalent modifiers, or light-reactive substructures such as rhodanines), or known assay-interfering structures (i.e., curcumins, polyphenols, catechols, quinones) and compound promiscuity, hit candidates were processed *in silico* through PAINS filters (32) and aggregation predictions using Badapple2 (33). Compounds displaying measurable activity against unrelated viruses (GAP-1081237), cell line-specific activity (GAP-1027282, GAP-1022901), lack of potency for sourced material (GAP-1027276, GAP-1000864), or selectivity indices <10 (CC_50_/EC_50_) (GAP-1041908) were discontinued.

### HTS screening libraries and primary hit compounds

HTS screening libraries were purchased from ChemDiv Inc. and the Chembridge Corporation. All libraries were curated to remove assay-interfering and undesirable chemical structures (32). ChemDiv libraries were selected from the ChemDiv Representative Diversity sets, which have been extensively filtered to remove reactive oxygen species and pan-assay interfering structures. The ChemDiv libraries were also further curated for drug-like and physicochemical properties, such as logP (mean = 3–4) and molecular weight (mean = 397). Chembridge libraries were selected from their CORE library stocks, including DIVERSet-CL and CNS-MPO subsets. Chembridge libraries were filtered to remove PAINS and possessed an average molecular weight and clogP of 337 and 1.5, respectively.

Primary screening hit candidates were sourced from their respective vendors to confirm screening activity and perform additional 96-well dose-response assays to determine EC_50_ and CC_50_ values. Compounds GAP-1358694 (cat #:9280022), GAP-1357119 (cat #:9248819), GAP-1356947 (cat #:9245804), GAP-1354666 (cat #:9205811), GAP-1350926 (cat #:9201987), and GAP-1173149 (cat #:73372258) were purchased from the Chembridge Corporation (https://www.hit2lead.com/). Compounds GAP-1146924 (cat #: Y504-6819), GAP-1081237 (cat #: L871-0191), GAP-1066658 (cat #: G868-0058), GAP-1041908 (cat #: E677-2070), GAP-1027282 (cat #: D053-0355), GAP-1027276 (cat #: D053-0308), GAP-1022901 (cat #: C618-0351), GAP-1022776 (cat #: C611-0649), and GAP-1000864 (cat #:3129-4934) were purchased from ChemDiv Inc. (https://www.chemdiv.com/). Compounds sourced from vendors were used for data shown in Fig. 1 and 3.

### CHIKV replicon assay

CHIKV replicons utilized a nLuc-PEST reporter fused in frame with the C-terminal domain of nsP3. Puromycin N-acetyltransferase was expressed under the control of the CHIKV subgenomic promoter. Stable replicon cell lines were generated by U-2-OS cells with *in vitro* transcribed RNA. Cells were incubated in puromycin-free medium for approximately 48 days prior to puromycin selection. Selection was performed with 1 µg/mL puromycin for 10 days. Replicon cell lines were maintained using complete media supplemented with puromycin (0.8 µg/mL).

### Time-of-addition studies

To identify the stage in the virus life cycle inhibited by each compound, time-of-addition assays were performed (59–64). U-2-OS cells were inoculated (MOI = 0.4 TCID_50_ units per cell) with CHIKV-nLuc. At the specified time points relative to infection, the indicated compounds were added to the culture media at a final concentration of 20 µM. Ammonium chloride was used at a concentration of 20 mM in cell culture media. Reporter gene expression was measured at 24 h after infection. Although multiple cycles of CHIKV replication can occur within a 24-h period, the inclusion of entry and polymerase inhibitors as internal controls validates the use of this ToA assay to determine whether compounds inhibit a stage of the viral life cycle after entry. The primary role of ToA assays was to determine whether inhibitory profiles of hit compounds were similar to entry inhibitors or if inhibition occurred post-entry.

### Focus forming assay

To quantify virus titers *in vitro* assays, U-2 OS cells seeded at 15,000 per well in 96-well plates were inoculated with 10-fold serial dilutions of virus prepared in Med-A (minimum essential medium supplemented with 0.2% BSA and 10 mM HEPES) for 2 h. After infection, the cells were overlaid with 1% carboxymethylcellulose in modified Eagle’s medium containing 2% FBS and 10 mM HEPES (pH 7.4). At 18 hpi, the cells were fixed by adding 100 µL of warm 1% paraformaldehyde (PFA; Electron Microscopy Science) in PBS and incubating for 1 h. Following five washes with PBS, the cells were permeabilized using 0.1% saponin in PBS with 0.1% BSA. For immunostaining, CHIKV-, SFV-, ONNV-, and MAYV-infected cells were incubated with a rabbit pAb to E2/E1 (65), RRV-infected cells with a rabbit polyclonal antibody against SFV capsid (66), and SINV-infected cells with monoclonal antibodies R2 and R6 targeting SINV E1 and E2 (67). Subsequently, cells were treated with horseradish peroxidase-conjugated goat anti-mouse or anti-rabbit IgG. Foci were visualized using TrueBlue Peroxidase substrate (#5510-0030, Seracare) and quantified with an ImmunoSpot S6 Macroanalyzer (Cellular Technologies).

### Dose-response antiviral assays

For CHIKV-nLuc–based dose-response assays, NHDF cells were seeded at 10,000 cells per well in 24-well plates and incubated for 24 h under standard culture conditions. Cells were subsequently inoculated with the indicated virus at MOI = 0.1 FFU per cell using Med-A medium for 1 h. Following infection, cells were washed three times with complete growth medium to eliminate unbound virus, and then treated with threefold serial dilutions of compounds. Cells were lysed at 24 hpi, and nanoluciferase activity was measured using the Nano-Glo Dual-Luciferase Reporter Assay System (Promega) on a PerkinElmer Victor X5 multilabel plate reader. Dose-response data were analyzed by non-linear regression with a variable slope model to determine EC_50_ using GraphPad Prism 10. For virus yield–based dose-response analyses, U-2 OS cells were seeded at 75,000 cells per well in 24-well plates, inoculated with the indicated alphaviruses, and treated with compounds as described above. At 24 hpi, supernatants were collected, clarified by centrifugation, and viral titers were determined using FFA. DMSO-treated cells were included as negative controls, and nanoluciferase signals were normalized to the respective DMSO control values. Dose-response data were analyzed by normalized non-linear regression with a variable slope model to determine EC_50_ using GraphPad Prism 10.

### Selection for viral resistance

U-2 OS cells were seeded at a density of 200,000 cells per well in six-well plates, cultured for approximately 24 h, and then inoculated with CHIKV primary stock at MOI = 1 FFU/cell. After 2 h, media containing compound or DMSO were added to three wells. At 16 hpi, supernatants were collected, clarified by centrifugation, frozen, and viral titers were determined using FFA. The concentration of compounds in each passage is shown in Fig. 6B. Viruses from P6 were plaque-purified, amplified in culture, and used for RNA isolation. Viral RNAs were reverse-transcribed and subjected to whole-genome sequencing as below. For competition studies, 1:1 mixtures (P1) of WT and mutant viruses were serially passaged three times on U-2 OS cells. Viral RNAs were isolated from each passage stock, and sequence analysis was performed as above to determine the ratio of WT vs. mutant at each passage.

### Whole-genome sequencing and data analysis

Viral whole-genome sequencing of CHIKV samples was performed using a metagenomic next-generation sequencing approach, as previously described (68). Libraries were sequenced on NextSeq 2000 with 2 × 150 bp read format. Raw reads were trimmed and quality filtered with fastp (v0.23.4) (69) (--cut_mean_quality 20 --cut_front --cut_tail --length_required 20 --low_complexity_filter --trim_poly_g --trim_poly_x). Filtered reads were then used for variant calling using the RAVA workflow (default parameters) and the CHIKV 181-Clone 25 (MK028839.1) as a reference (https://github.com/greninger-lab/RAVA_Pipeline/tree/2025-08-14_AECM_MK-PY_C3-4_CHIKV_publication) (70, 71).

### Cytotoxicity assay

NHDF cells were seeded at 10,000 cells per well in 24-well plates and cultured for 24 h. Cells were then treated with threefold serial dilutions of compounds starting at 500 µM and incubated for 24 h. DMSO was included as a negative control for normalization. Following treatment, cells were incubated with PrestoBlue reagent (ThermoFisher Scientific) for 1 h at 37°C, and fluorescence was measured using a PerkinElmer Victor X5 multilabel plate reader. The CC50 values were determined by normalized non-linear regression with a variable slope model in Prism (GraphPad).

### Trans-replication assays

U-2 OS cells were seeded at 75,000 cells per well in 24-well plates and cultured for 24 h. Cells were then co-transfected with 250 ng of plasmid encoding template RNA and 250 ng of plasmid encoding P1234 WT (72, 73), using Lipofectamine LTX with PLUS reagent (ThermoFisher Scientific) following the manufacturer’s instructions. After 4 h of post-transfection, medium containing 100 µM of compounds or DMSO was added to the cells. Cells were then incubated for 20 h, lysed, and GLuc activity was quantified using the Dual-Luciferase Reporter Assay System (Promega). Luciferase activities were normalized to those of the DMSO-treated controls.

### *In silico* docking

Docking studies were performed with MOE 2024.06. The ONNV nsP4 structure (PDB 7Y38) was used for docking studies. After protonation and energy minimization, an induced-fit protocol was used to dock GAP-1146924 and GAP-1173149 into the ONNV nsP4 structure based on resistance data information. To reduce the likelihood of erroneous docking poses, the mutation capable of conferring reduced susceptibility to each antiviral hit was chosen as a target. For GAP-1146924, residue Q542 was selected as the target site for docking. For GAP-1173149, residue A350 was selected as the target site for docking. Initial placement was performed using the Triangle Matcher routine using London dG scoring. Post-placement refinement for the top 30 preliminary docking poses was refined using an Induced Fit protocol (GBVI/WSA dG scoring). The top-scoring docking pose for each compound is shown in Fig. 8. Summaries of the *in silico* docking scores are shown in Table 3.

### Synthesis of 2-(2,5-dioxoimidazolidin-1-yl)-N-(1,4,5,6,7,8-hexahydrocyclohepta[d]pyrazol-3-ylmethyl)acetamide (GAP-1173149)

2-(2,5-dioxoimidazolidin-1-yl)acetic acid was dissolved in 30 mL DMF and to the reaction added 5.06 g (13.31 mmol 1.1 eq) HATU and the reaction mixture was brought to 0°C. Then, the amine 1,4,5,6,7,8-hexahydrocyclohepta[d]pyrazol-3-ylmethanamine (2 g, 12.1 mmol) and 2.11 mL (12 mmol, one eq) DIPEA were added and stirred while letting the mixture come to room temperature for 6 h. After checking reaction completion by thin-layer chromatography and staining with PMA, and LC-MS, the reaction was terminated by the addition of a few drops of HCl solution, and extracted two times with cold water and ethyl acetate. The organic layers were collected, dried with magnesium sulfate, filtered, and evaporated. The residue was purified using column chromatography with DCM and an increasing ratio of DCM:MeOH:ammonia (9:1:0.2) mixture and then with reverse phase using the C18 reverse phase column water/acetonitrile system to yield 1.3 g 2-(2,5-dioxoimidazolidin-1-yl)-N-(1,4,5,6,7,8-hexahydrocyclohepta[d]pyrazol-3-ylmethyl)acetamide (GAP-1173149) with 35% yield. GAP-1173149, synthesized in-house, was used to collect all data shown in Fig. 4 through 7.

### Synthesis of 2-(4-fluorophenyl)-N-[(2-methylpyrazol-3-yl)methyl]ethanamine Intermediate

2-(4-fluorophenyl)ethanamine (1 g, 7.19 mmol) was dissolved in anhydrous DCM. 2-methylpyrazole-3-carbaldehyde (791 mg, 7.19 mmol, 1 eq) and titanium (IV) isopropoxide (2.53 mL, 8.62 mmol, 1.2 eq) were added to the reaction and stirred under an argon atmosphere for 6 h. After this time, 3.81 g (17.96 mmol, 2.5 eq) of sodium triacetoxyborohydride and a catalytic amount of acetic acid were added and stirred overnight. The reaction was then brought to pH 10 with NaOH solution. More DCM and water were added. Phases separated, organic layers collected, dried with magnesium sulfate, filtered, and evaporated under vacuum. Products were purified using flash chromatography with hexanes and an increasing ratio of ethyl acetate to obtain 1.24 g 2-(4-fluorophenyl)-N-[(2-methylpyrazol-3-yl)methyl]ethanamine with 74% yield.

### Synthesis of N-[2-(4-fluorophenyl)ethyl]-1,5-dimethyl-N-[(2-methylpyrazol-3-yl)methyl]pyrazole-4-sulfonamide (GAP-1146924)

2-(4-fluorophenyl)-N-[(2-methylpyrazol-3-yl)methyl]ethanamine (1.24 g, 5.32 mmol) was dissolved in 20 mL anhydrous THF, and N,N-DIPEA (1.85 mL, 10.63 mmol, 2 eq) was added. The reaction was brought to 0°C, and then 1.14 g (5.85 mmol, 1.1 eq) of 1,5-dimethylpyrazole-4-sulfonyl chloride was added. The reaction was allowed to warm up to room temperature and stirred for 3 h in total. After the reaction was complete, ethyl acetate and water were added for extraction. Organic layers were collected, dried using magnesium sulfate, filtered, and evaporated under vacuum. Products were purified using flash chromatography with hexanes and an increasing ratio of ethyl acetate to obtain 1.2 g N-[2-(4-fluorophenyl)ethyl]−1,5-dimethyl-N-[(2-methylpyrazol-3-yl)methyl]pyrazole-4-sulfonamide (GAP-1146924) with 60% yield. GAP-1146924, synthesized in-house, was used to collect all data shown in Fig. 4 through 7.

### NMR analysis of GAP-1173149

^1^H NMR (400 MHz, MeOD) δ 4.34 (s, 2H), 4.13 (s, 2H), 4.01 (s, 2H), 2.74–2.67 (m, 2H), 2.59–2.49 (m, 2H), 1.85 (qd, *J* = 6.1, 3.3 Hz, 2H), 1.71–1.59 (m, 4H). (NH protons exchanged with MeOD). ^13^C NMR (151 MHz, MeOD) δ 172.48, 167.28, 158.10, 147.99, 142.30, 117.87, 46.20, 39.88, 34.11, 31.90, 28.71, 27.31, 27.28, 23.62.

### NMR analysis of 2-(4-fluorophenyl)-N-[(2-methylpyrazol-3-yl)methyl]ethanamine Intermediate

^1^H NMR (400 MHz, CDCl3) δ 7.39 (d, *J* = 1.8 Hz, 1H), 7.21–7.12 (m, 2H), 7.06–6.92 (m, 2H), 6.13 (d, *J* = 1.8 Hz, 1H), 3.84 (s, 3H), 3.82 (s, 2H), 2.90 (t, *J* = 1.1 Hz, 2H), 2.80 (t, *J* = 6.9 Hz, 2H), 1.82 (s, 1H). ^19^F NMR (376 MHz, CDCl3) δ −116.97.

### NMR analysis of GAP-1146924

^1^H NMR (400 MHz, DMSO) δ 7.81 (s, 1H), 7.37 (d, *J* = 1.8 Hz, 1H), 7.10–6.95 (m, 4H), 6.28 (d, *J* = 1.8 Hz, 1H), 4.35 (s, 2H), 3.79 (s, 3H), 3.75 (s, 3H), 3.20–3.10 (m, 2H), 2.52–2.49 (m, 2H), 2.44 (s, 3H). ^19^F NMR (376 MHz, DMSO) δ −116.80. ^13^C NMR (101 MHz, DMSO) δ 161.31 (d, *J* = 241.7 Hz, C-F), 141.01, 138.60, 137.91, 137.04, 134.85 (d, *J* = 3.1 Hz, C-C-C-C-F), 130.72 (d, *J* = 8.0 Hz, C-C-C-F), 116.61, 115.49 (d, *J* = 21.1 Hz, C-C-F) 107.73, 49.51, 43.32, 37.21, 36.72, 34.18, 10.29.

### Statistics and reproducibility

CBIS, GraphPad Prism (10.3.0), and Excel (16.92) software packages were used for data analysis. Statistical significance was assessed using ordinary one-way ANOVA with multiple comparisons test in GraphPad Prism. For antiviral potency and cytotoxicity measurements, effective concentrations were determined from dose-response data sets through four-parameter variable slope regression modeling. Biological repeats indicate measurements taken from distinct samples.

## Data Availability

CHIKV whole-genome sequencing data of the compound-treated and DMSO-treated passages are available in NCBI BioProject PRJNA1306170. HTS and dose-response raw data are available from the corresponding authors upon request. Numerical data for all biological experiments associated with the individual figures are available from the corresponding authors upon request. However, no chemical structure information concerning the composition of screening libraries and unsuccessful hit candidates will be provided. Homology modeling software is available from the AlphaFold modeling server at https://alphafoldserver.com/. All other software solutions employed are commercially available.
